# Adverse childhood experiences, bullying, inflammation and BMI in 10-year-old children: The biological embodiment

**DOI:** 10.1371/journal.pone.0273329

**Published:** 2022-08-19

**Authors:** Sara Soares, Ana Cristina Santos, Sílvia Fraga

**Affiliations:** 1 EPIUnit—Instituto de Saúde Pública, Universidade do Porto, Rua das Taipas, Porto, Portugal; 2 Laboratório para a Investigação Integrativa e Translacional em Saúde Populacional (ITR), Rua das Taipas, Porto, Portugal; 3 Departamento de Ciências da Saúde Pública e Forenses e Educação Médica, Faculdade de Medicina, Universidade do Porto, Porto, Portugal; University of South Florida, UNITED STATES

## Abstract

Exposure to adversity during the first years of life might already be biologically embedded well before adult life. Thus, the impact of different stressful experiences needs to be explored. This study aims to examine if the association between being victimized (adverse childhood experiences—ACEs and bullying) and (hs-) C-Reactive Protein (CRP) is explained by the influence of adversity on the body mass index (BMI) of the child. We included children from the Portuguese birth cohort Generation XXI (n = 3712) that at 10 years of age completed a questionnaire on the exposure to ACEs and bullying victimization, assessed by an adaptation from the original ACEs study and an adaptation of The Bully Scale Survey, respectively. Following an overnight fast, a venous blood sample was collected by trained nurses and hs-CRP was assayed in fresh blood samples. Weight and height were measured with the child in underwear and bare feet. Weight was measured to the nearest one-tenth of a kilogram with the use of a digital scale (Tanita), and height was measured to the nearest one-tenth of a centimetre with the use of a wall stadiometer (seca®). BMI was calculated as the value of weight (kg) over squared height (m), and computed as an age- and sex-specific BMI standard deviation (SD) score (z-score), according to the World Health Organization Child Growth Standards (5–19 years). Regression coefficients and respective 95% Confidence Interval [β(95%CI)] were computed using path analysis. We observed that ACEs had a positive total effect on hs-CRP at the age of 10 years (β = 0.06; 95%CI: -0.02; 0.15). A direct effect (β = 0.02; 95%CI: -0.01; 0.06) accounted for 66.1% of the association between ACEs and hs-CRP. A positive total effect of bullying victimization on hs-CRP (β = 0.20; 95%CI: 0.06; 0.34) was observed. A direct effect (β = 0.08; 95%CI: -0.05; 0.21) accounted for 40.0% of the association, while an indirect effect through BMI (β = 0.12; 95%CI: 0.06; 0.18) explained 60.0% of the pathway between bullying victimization and hs-CRP. Results suggest that there might be different mechanisms involved in the biological embedding of childhood experiences. BMI seems to explain a great part of the association between exposure to bullying victimization and hs-CRP at 10 years of age. Further research is still needed to better understand the mechanisms explaining the emergence and persistence of health poorer outcomes later in life for victims of abuse.

## Introduction

Adverse childhood experiences (ACEs) are defined as stressful and/or traumatic experiences that occur during childhood and include emotional, physical, and sexual abuse, emotional and physical neglect, and household dysfunction [1]. ACEs have been associated with a life-long increased risk of developing cardiovascular disease, cancer, among others, and even premature death [1–3]. These negative exposures have a broad impact on child development [4, 5] and are also associated with the occurrence of mental health problems later in life [4, 6], accounting for 29.8% of all psychiatric disorders [7]. Even though exposure to adversity may have a long-lasting and cumulative health effect, these experiences seem to have an immediate impact on the child’s health.

While ACEs include experiences occurring in the household, bullying experiences, more common at school, have also been recognized as an ACE [8, 9]. Similarly to other ACEs, bullying in childhood has also a great impact on mental and physical health [10, 11]. Victims of bullying (such as having belongings taken or having been hit or beaten up) present increased behavior problems, hyperactivity and conduct problems compared to those not involved [12], during childhood, and suicidal ideation/attempts in adolescence [13]. Also, victims of bullying during childhood have worse health outcomes [14], poorer perceived quality of life [15] and increased risk of psychiatric disorders [16] and suicidality [15] in adult life. Even though the biological consequences of bullying in childhood have been poorly studied [17], victims present increased levels of C-Reactive protein (CRP) levels in adolescence [17] and during mid-life [18]. Also, some specificity regarding the type of involvement in this behavior since the involvement as a victim or as a bully could be related to different effects on biological markers [17].

The link between exposure to ACEs and/or bullying and health outcomes later in life may be explained by the inflammatory process [19–21], and repeated exposures to adversity are likely to affect the human stress regulatory system, followed by an increase in inflammatory levels [22, 23]. Once the detrimental effects of adversity on the human stress response system are in place, they will result in chronic inflammation over the life course [24, 25]. However, the association between adversity and disease might be explained through the adoption of unhealthy behaviors (e.g., poor diet, sedentary behavior, smoking) [26, 27], or via stress sensitization, in particular through the hypothalamic-pituitary-adrenal (HPA) axis [23]. The prolonged HPA axis activation may lead to altered insulin sensitivity, increased blood pressure, and inflated central adiposity, consequently leading to elevated low-grade inflammation [23, 28, 29] and high levels of CRP [30]. Even though the biological mechanisms mediating these associations remain unclear, childhood adversity seems to contribute to a pro-inflammatory state in adulthood [31, 32], and some studies showed that body mass index (BMI) mediated the association between early-life adversity and inflammation in adolescence [33, 34].

Although the association of victimization with inflammation has been increasingly recognized, the embodiment might be operating differently with each adverse experience. Different pathways seem to be involved in the bodily incorporation of such experiences. The embodiment may be caused by victimization in the familial environment, such as ACEs; or victimization by bullying, more related to peer relationships and the school environment. Therefore, the association of victimization experiences with BMI still needs clarification. Literature has established that food is sometimes used as a coping mechanism in response to stress, commonly known as emotional eating, overeating [35] or selecting high-calorie foods when facing stressful circumstances [36], behaviors that will increase per cent body fat, overweight, and obesity. Sedentary behavior and/or poor diet may lead to increased BMI, explaining some of the association between victimization experiences and hs-CRP, via adiposity.

Given recent research implicating inflammation in promoting a variety of serious mental and physical health problems, using data from 10-year-old participants of the Portuguese birth cohort, Generation XXI, this study aims to examine if the association of being victimized (via exposure to ACEs or bullying) and CRP is explained by the influence of adversity in the BMI of the child.

## Materials and methods

### Study design and participants

The study sample consisted of children who participated in Generation XXI, a prospective Portuguese-population-based birth cohort. As previously reported [37], recruitment occurred during 2005–2006. Briefly, mothers and children (n = 8647) were recruited in public maternity units in Porto, Portugal. The entire cohort was invited to attend the second (2009–2011), the third (2012–2014) and the fourth (2016–2017) study waves, when children were aged four, seven and ten years old, respectively. Anthropometric measures and blood samples were collected in all study waves, following the same standardized procedures. Data on demographic and socioeconomic characteristics, personal history of disease and health-related behaviors were collected by trained interviewers through structured questionnaires. Generation XXI was approved by the Portuguese Data Protection Authority and by the Ethics Committee of Hospital São João, and data confidentiality and protection were guaranteed in all procedures according to the Declaration of Helsinki. Signed informed consent was obtained for all adults and children participants had it signed by their legal guardian at every study wave [37]. Generation XXI uses a split-file anonymisation method to maintain confidentiality. Participants within each data collection wave are assigned a unique identifier, and investigators work with anonymised data and do not have access to identifiable information.

The present investigation includes data on participants with complete information on ACEs and bullying victimization reports and on hs-CRP levels at the age of 10 years. Thus, the analyses were based on data from 3712 participants from the study’s fourth wave, and the sample description is presented in Table 1.

**Table 1:** Child’s, family, and parental characteristics.

	Total, n (%)
*Child’s characteristics*	
Sex	
Female	1772 (47.7)
Male	1940 (52.3)
Age (years)	
Mean (SD)	10.1 (0.33)
Weight (Kg)	
Mean (SD)	37.7 (8.74)
Height (m)	
Mean (SD)	1.41 (6.56)
BMI*	
Underweight/ Normal	2130 (57.4)
Overweight/ Obese	1582 (42.6)
CRP	
Median (P25-75)	0.5 (0.2–1.3)
*Exposure to adverse childhood experiences*	
Bullying victimization	
No	3318 (89.7)
Yes	379 (10.3)
ACEs	
0–3	2119 (57.1)
4 or more	1593 (42.9)

ACEs: Adverse childhood experiences; BMI: body mass index; CRP: high sensitivity C-Reactive Protein

*BMI z-score, age and sex-specific BMI standard deviation scores according to the World Health Organization (WHO, 2006)

### Measures

#### ACEs

Conventional ACEs questions, adapted from the original CDC-Kaiser ACE study [1] and ACEs questions adapted from the Child and Adolescent Survey of Experiences: Child Version (CASE) [38] were used to collect information through self-administered questionnaires and children were helped by a trained interviewer whenever requested. Children reported their lifetime experience of moving from a house, school or neighbourhood against their will; learning problems at school; the death of a family member; injury or serious illness in the family; child hospitalization due to a disease or an accident; parents called to school because the child was in trouble; parental divorce or separation; financial issues in the household; a family member with a drug or alcohol addiction; incarceration of a household member; witnessing parents arguing or fighting; experiencing someone in the household shouting, yelling or screaming; insulting or humiliating the child; and finally, being hit, kick or punched by someone at home. For each item children could choose “yes” or “no”, as if the adversity had happened to them or not, respectively. The score of ACEs considered the sum of the number of exposures and was defined as having up to 3 experiences and having 4 or more experiences.

#### Bullying

The Bully Scale Survey, a structured questionnaire created by the Centers for Disease Control and Prevention [39] was used to assess bullying involvement. In a private setting, and after the specific parents’ consent on information collection, children reported the frequency (never, rarely, sometimes, often, and always) in which they were involved in bullying either as a victim and/or as an aggressor.

Victims of bullying were defined as those who answered “always” to one or more: How did you get bullied? (Check how often this happened) *“Called me names”*, *“Made fun of me”*, *“Said they will do bad things to me”*, *“Played jokes on me”*, *“Won’t let me be a part of their group”*, *“Broke my things”*, *“Attacked me”*, *“Nobody would talk to me”*, *“Wrote bad things about me”*, *“Said mean things behind my back” “Pushed or shoved me*”; and mentioned to have never been aggressors of bullying.

#### Hs-CRP

Following an overnight fast, a venous blood sample was collected before 11 a.m. by trained nurses in our research centre, after applying a topical analgesic cream (EMLA cream). The samples were centrifuged at 3500 rpm for 10 min and plasma was aliquoted. Hs-CRP was assayed in fresh blood samples using CardioPhase®hsCRP Flex®, Dimension Vista® System, from Siemens. All blood evaluations were performed at the Clinical Pathology Service, São João Hospital Center, Porto, Portugal. The participants below the minimum detection limit for CRP were assigned values of 0.02 mg/l [40].

#### BMI

Trained technicians performed anthropometric measurements according to standardized procedures. In brief, weight and height were measured with the child in underwear and bare feet. Weight was measured to the nearest one-tenth of a kilogram with the use of a digital scale (Tanita), and height was measured to the nearest one-tenth of a centimetre with the use of a wall stadiometer (seca®). BMI was calculated as the value of weight (kg) over squared height (m), and computed as an age- and sex-specific BMI standard deviation (SD) score (z-score), according to the World Health Organization Child Growth Standards (5–19 years) [41]. In the models, BMI was used as a continuous variable, and recoded into underweight or normal weight (BMI <2 SD) and overweight/obese (BMI >2 SD), for descriptive purposes.

### Data analysis

Pearson correlations between age, sex, parental education, ACEs exposure, bullying victimization, BMI and hs-CRP were computed. Due to a highly skewed distribution, and for statistical purposes, hs-CRP was log-transformed. Also, high levels of hs-CRP that could represent an acute condition rather than a chronic inflammatory state [42], lead to the exclusion from the analyses of participants with hs-CRP levels higher than 10 mg/L (n = 85).

**Fig 1:**
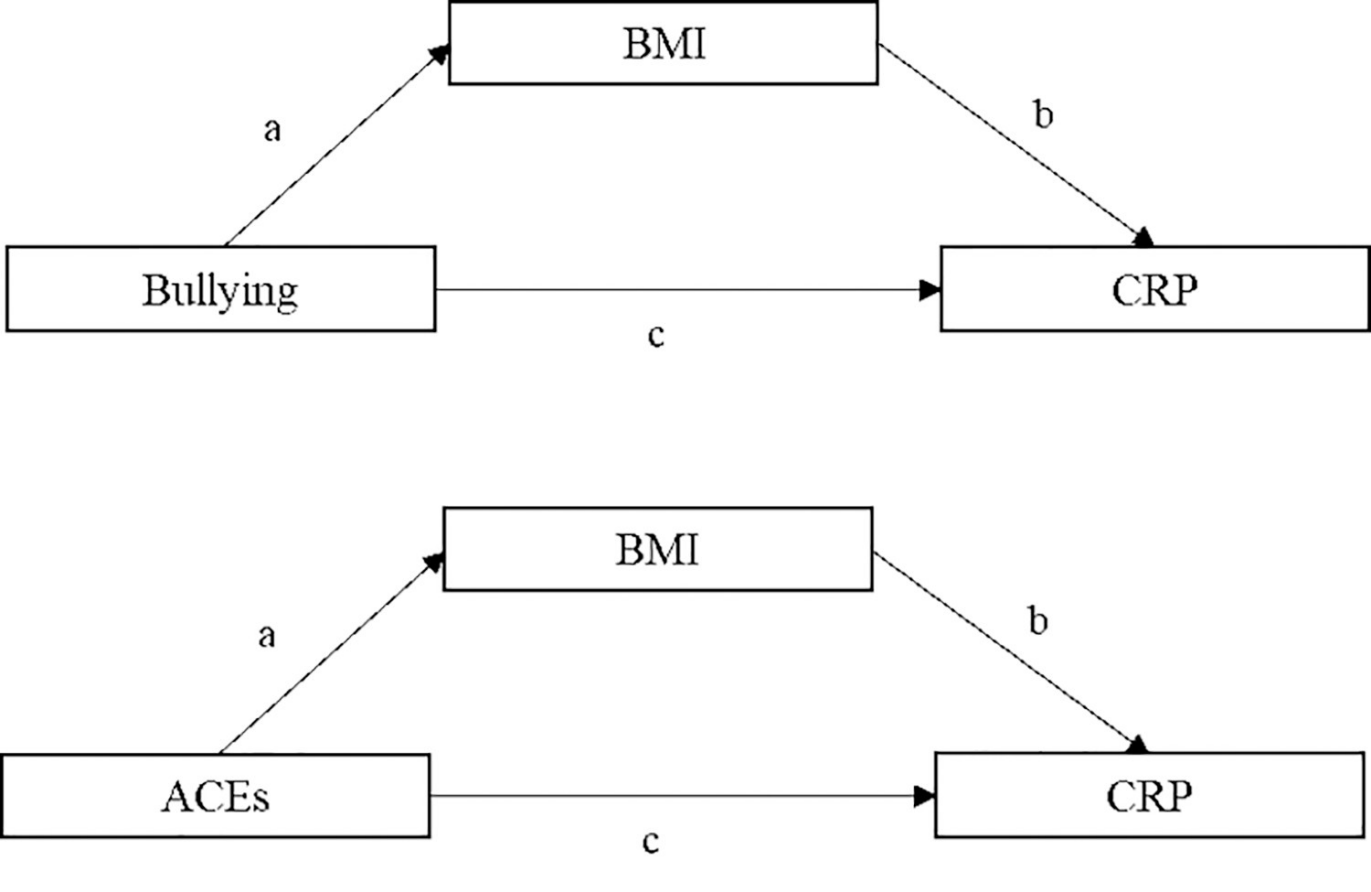
Conceptual framework of the mediation models for the present study. Indirect effect = ab, direct effect = c, total effect = ab + c.

Path analysis was used to estimate the regression coefficients (β) and 95% confidence intervals (95% CI), which represent the increase in mg/L of log hs-CRP levels with exposure to ACEs and bullying victimization. Path analysis was conducted based on the theoretical model depicted in Fig 1. The presence of multicollinearity was assessed by calculating the Variance Inflation Factor (VIF), which was lower than 5, indicating no multicollinearity [43]. Bootstrapping was used for the estimation of the 95% CI for the direct and indirect effects. The Comparative Fit Index (CFI) [44], the Tucker–Lewis Index (TLI) [45], and the Root Mean Square Error of Approximation (RMSEA) were used to assess the general fit of the models[46]. A CFI and TLI equal to or higher than 0.90 [47] and a RMSEA lower than 0.05 indicate a good model fit [48]. Analyses were performed using STATA version 15.1.

In the models, sex and parental education were included as covariates. Parental education was measured as the number of years of formal schooling completed and classified according to the International Standard Classification of Education 2011 classes [49]. The low educational level corresponded to 9 years or less of formal schooling; intermediate education to 12 years of formal education; and high education to more than 12 years of formal education.

## Results

Table 1 shows the participants’ characteristics. In this sample, more than half were male (52.3%) and their mean (SD) age at Generation XXI’s fourth wave was 10.1 (0.33) years. Participants’ mean (SD) weight was 37.7 (8.74) kg and most of them were classified as being underweight/normal BMI (57.4%). The median of hs-CRP levels was 0.5 (P25-75: 0.2–1.3) mg/L. Bullying victimization was reported by 10.3% of participants and 42.9% of the children reported four or more ACEs.

Correlations between all study variables are presented in Table 2. Positive, but weak, correlations were observed for ACEs and BMI (r = 0.08) and hs-CRP (r = 0.02). Bullying victimization was positively correlated with BMI (r = 0.08) and hs-CRP (r = 0.04).

**Table 2:** Pearson correlations between all the variables included in the models.

	Age	Sex	Parental education	ACEs	Bullying	BMI	hs-CRP
Age	1.00						
Sex	-0.02	1.00					
Parental education	-0.07*	0.02	1.00				
ACEs	0.02	0.07*	-0.08*	1.00			
Bullying	0.03	0.08*	-0.06*	0.23*	1.0		
BMI	-0.01	0.02	-0.10*	0.08	0.08*	1.00	
hs-CRP	-0.02	-0.09*	-0.07*	0.02	0.04**	0.44*	1.00

*p<0.01

**p<0.05

ACEs: Adverse childhood experiences; BMI: body mass index; hs-CRP: high sensitivity C-Reactive Protein.

A positive total effect of ACEs on hs-CRP levels (β = 0.06; 95%CI: -0.02; 0.15) was estimated. In this model, and independently of the child’s sex, a direct effect (β = 0.02; 95%CI: -0.01; 0.06) and an indirect effect through BMI (β = 0.04; 95%CI: -0.03; 0.12) were observed. The indirect effect explains 33.9% of the pathway between ACEs and hs-CRP. After additional adjustment for parental education, the indirect effect between ACEs and hs-CRP accounted for 34.9% of the pathway (Table 3).

**Table 3:** Total, direct and indirect effects derived from the path analysis model for the association between ACEs, bullying and BMI and CRP.

	hs-CRP	% of the total effect
	β (95%CI)	
*ACEs* ^1^		
Total effect on hs-CRP	0.06 (-0.02; 0.15)	
Direct effect on hs-CRP	0.02 (-0.01; 0.06)	66.1
Indirect effect through BMI	0.04 (-0.03; 0.12)	33.9
*ACEs* ^2^		
Total effect on hs-CRP	0.04 (-0.04; 0.13)	
Direct effect on hs-CRP	0.01 (-0.02; 0.05)	65.1
Indirect effect through BMI	0.03 (-0.05; 0.11)	34.9
*Bullying* ^1^		
Total effect on hs-CRP	0.20 (0.06; 0.34)	
Direct effect on hs-CRP	0.08 (-0.05; 0.21)	40.0
Indirect effect through BMI	0.12 (0.06; 0.18)	60.0

^1^adjusted for sex

^2^adjusted for sex and parental education; β(95%CI): Beta and corresponding 95% confidence interval; ACEs: Adverse childhood experiences; BMI: body mass index; hs-CRP: high sensitivity C-Reactive Protein

Additionally, a positive total effect of bullying victimization on hs-CRP levels (β = 0.20; 95%CI: 0.06; 0.34) was estimated. In this model, and independently of sex, a direct effect (β = 0.08; 95%CI: -0.05; 0.21) and an indirect and statistically significant effect through BMI (β = 0.12; 95%CI: 0.06; 0.18) were observed. The indirect effect explains 60.0% of the pathway between bullying victimization and hs-CRP (Table 3).

The formal analysis found no interaction between sex and ACEs and bullying victimization.

## Discussion

In a sample of participants of the Generation XXI birth cohort, we observed that childhood BMI played a pivotal role in the pathway from bullying victimization to inflammation, while the association between adverse experiences in the family context and inflammation seemed to be less mediated by BMI. Despite well-documented associations between early life adversity and later inflammation, less is known about how such experiences exert effects on inflammation in childhood and if increased BMI plays a role in this association. The present study focused on early life adversity within a family context and peer-related victimization and their relationships to BMI to investigate the links between adversity in infancy and low-grade, chronic inflammation.

Identifying which mechanism is responsible for the increase in inflammation levels and consequently the establishment of low-grade inflammation is of utmost importance to define effective strategies to prevent and mitigate the health-related consequences of ACEs later in life. Also, it is described that living in an environment of exposure to ACEs (stem from research conducted by the CDC and Kaiser Permanente Adverse Childhood Experiences Study) may be closely related to growing with diminished or unpredictable household availability of sufficient, adequate and nutritious food [50]. In the model with ACEs, it was observed that the direct effect accounted for most of the association with hs-CRP. The indirect effect of a high number of adverse experiences in the household on the hs-CRP levels through BMI accounted for 33.9% of the association studied. Thus, the direct effect seemed to account for most of this association, meaning that this effect may be more related to the HPA axis regulation rather than to the adoption of health-risk behaviors. As the majority of association occurred directly on hs-CRP, this might be explained by the variation in the HPA axis activity commonly associated with the neurobiology of stress sensitization [23, 51], leading to altered insulin sensitivity, increased blood pressure, inflated central adiposity, and consequently to elevated low-grade inflammation [23, 28] and high levels of CRP [23, 28, 30]. Also, as some evidence has been published on the causal relations between socioeconomic circumstances and exposure to ACEs [52], we investigated the role of parental education on this association and observed that the association remained similar.

On the contrary, the direct effect of bullying victimization on hs-CRP levels was lower than the BMI-mediated effect, accounting for 60.0% of the association. Thus, most of the association between bullying victimization and hs-CRP is likely to be driven by BMI. Even though some discussion remains on the mechanisms that might be involved in the biological embodiment of these experiences [51, 53], which is potentially different from the ones involved in ACEs, our results on the mediation effect of BMI showed that health behaviors seemed to be important in the establishment of chronic low-grade inflammation, at least, at a shorter-term.

These results may be reflecting the existence of different pathways that explain adversity embodiment, whether it is caused by victimization through exposure to adverse experiences more related to the familial environment, or victimization by bullying, more related to peer relationships and school environment. Also, these results reinforce the importance of health risk behaviors in the embodiment of adversity. We may speculate that exposure to bullying might lead to the adoption of negative health behaviors [54, 55], as a way of trying to reduce tension or stress with a potential contribution to later disease development [1, 2] or premature death [3]. Higher inflammation levels have been observed in participants reporting bullying victimization when compared with participants non-victims, and with aggressors [17]. Even though we do not expect the same contribution for all health-related behaviors, such as smoking or alcohol consumption, as they are not expected to be established in children, sedentary behavior and/or poor diet may lead to increased BMI, explaining some of the association between bullying and hs-CRP, via adiposity. Especially when recent literature has established that food is sometimes used as a coping mechanism in response to stress, commonly known as emotional eating, overeating [35] or selecting high-calorie foods when facing stressful circumstances [36], behaviors that will increase per cent body fat, overweight, and obesity.

Two studies showed that increased BMI mediated the association between early-life adversity and inflammation in adolescence [33, 34]. Our results add to these demonstrating, to a certain degree, a role of adiposity in the studied association. One of the studies found that BMI mediated the association between serious interpersonal conflict stress and increased hs-CRP, but no association was observed regarding serious financial stress or maternal depression [33], while the other study showed that BMI attenuated the associations between cumulative adverse events and immediate events, that included being taken into foster care, being a victim of physical or sexual abuse or separated from mother or father, and inflammation [34]. However, neither quantified the indirect effect of BMI in the studied associations.

It is important to emphasize that higher levels of inflammation do not necessarily mean risk, even though, children’s inflammatory profile may contribute to the earlier establishment of an unfavorable profile in early life stages. However, we cannot assume a deterministic view and conclude that these children will develop disease later in life. But it can be hypothesized that the underlying atherosclerotic process might already be in course and high levels of inflammatory markers at such early ages can be a precursor of later development of disease [23, 29]. And the results seemed to show a direct and indirect effect of traumatic experiences on hs-CRP levels, which may have the potential of accumulation by continuous exposure [56].

### Strengths and limitations

The use of data from the well-established population-based birth cohort, Generation XXI is one of the main strengths of this study. However, because it is common in prospective birth cohorts, there has been attrition over time, leading to a reduction in the sample size and a more socioeconomically advantaged group of participants throughout childhood. A comparative analysis was conducted between the group of participants who met inclusion criteria and those who did not for the present study and data indicated that participants who were not included in the study belonged to families with a lower monthly disposable household income and with lower parental education.

One of the main limitations of many papers published exploring the association between exposure to traumatic experiences and adult inflammation, is that authors are highly dependent on retrospectively collected data, and thus limited by recall bias. We overcame this limitation as our analysis used data on ACEs and bullying victimization collected at the age of 10 years, and by that less exposed to recall bias. Recent studies have described discordance in the ideal timing of the collection of information reporting of ACEs [57, 58]. We explored exposure to ACEs and how the child felt when the event occurred, involvement in bullying as a victim and the frequency by which the victimization occurred during the lifetime, all reported by the child. In regards to ACEs, we used a dichotomized variable that shows that those children are in fact in a situation of increased risk. Although only one adversity might impact children’s lives, the increasing number of adversities put children in a worse health trajectory [3, 59, 60] and consequently are the ones that already show biological alterations, such as in CRP [60]. Also, we used data from solely victims, excluding participants that may have been involved in bullying also as aggressors. This decision is supported by data reporting that being bullied predicted greater increases in CRP levels, whereas bullying others predicted lower increases in CRP compared with those not involved in bullying [17]. Exposures were collected close to the occurrence, and children described their own experiences in a safe and protective environment. Children exposed to ACEs are more frequently from less socioeconomic advantaged families [61], and, for that reason may be more exposed to environmental and physical risk factors, and consequently be more susceptible to infections [62]. Trying to minimize the effect of acute infections, we excluded participants with hs-CRP levels higher than 10 mg/L [42] from the analyses. This option is justified by other results showing significant short-term (approximately 2.5 weeks) within-person variability in CRP levels, particularly at high values. Approximately one-third of persons with elevated CRP levels were reclassified as having normal CRP levels after repeated testing [63]. Otherwise, this biomarker is relatively stable and one sample is sufficient to obtain a reliable level of hs-CRP in circulation [64]. Thus, although this study only comprised one measure of inflammation, the marker of systemic inflammation is widely used in several population studies [65, 66]. Hs-CRP levels have been successful in establishing an association between exposure to adverse events with prolonged low-grade activation of the immune system and consequently higher inflammatory levels [23, 29]. Moreover, the measure of inflammation was performed when children answered the questions on ACEs and bullying victimization.

As our sample was exclusively Caucasian, there is no ethnic variability to account for. Thus, ethnicity is not considered in the observed associations and was not taken into account in the models. Also, underweight children deserve further consideration as poor nutrition during adolescence and young adulthood implies poorer quality of life and additional health and morbidity risks [67]. This sensitivity analysis could not be conducted in our sample due to the small number of children within this group.

The present analyses are based on cross-sectional data and the cross-sectional study design limits the conclusions. So, conclusions can only be drawn regarding associations, but not about cause and effects, and to fully understand the proposed models, they should be tested in a longitudinal study. However, although these analyses were based on previous studies and follow similar methodological options [68–71], some controversy may arise regarding if bullying occurred after the increase in BMI. Moreover, when it comes to bullying, bullies are looking for a victim whom they can assert power over [72]. But their choice on who to bully is much more complex than picking on people weaker than them. There are a variety of reasons that a person might become a bullying victim, including everything from personality differences to being in the wrong place at the wrong time, poorer social and emotional adjustment, greater difficulties in making friends, poorer social relations with peers and more feelings of loneliness [73, 74]. Although a bulk of literature shows that overweight or obese adolescents were more at risk of being bullied, other authors also state that the more boys and girls reported negative feelings resulting from weight-based bullying, the more they also reported coping with these experiences by avoiding gym class, consuming more food, and binge eating [75]. Also, children who were chronically bullied in school were 1.7 times more likely to be overweight as young adults than non-bullied children and bullied children also had a higher BMI and waist-hip ratio at the age of 18. These associations were independent of other environmental risk factors and of their genetic risk of being overweight. Also, at the time of victimisation, bullied children were not more likely to be overweight than non-bullied children, indicating that overweight children were not simply more likely to fall victim to bullying [76]. Moreover, in our sample, we tested to see the direction of the association between BMI and bullying and observed that the association is stronger when BMI is considered as exposure. Finally, BMI at the age of 7 years was not associated with bullying victimization at the age of 10 years, when adjusted for BMI at 10 years. Nevertheless, future analyses should establish causal links or at least a time sequence of the studied effects.

### Future studies

Further research is required to measure ACEs dimensions and bullying experiences in different datasets and test their effects on more and repeated measures of biomarkers, released in response to trauma, inflammation, and infection. These should include interleukin-6 (IL-6), interleukin-1β (IL-1b), interleukin-17 (IL-17) and tumor necrosis factor-α (TNF-a). In addition, future studies should seek to determine how the interplay between ACEs and bullying and genetic factors might influence trajectories of biomarkers and other health outcomes across the life course, considering the behaviours acquired throughout adolescence.

## Conclusion

This study suggests that there might be different mechanisms involved in the biological embedding of childhood experiences. BMI seems to mediate most of the association between exposure to bullying victimization and hs-CRP levels at the age of 10 years. However, BMI does not seem to mediate the association between exposure to ACEs and hs-CRP levels.

Adverse experiences in childhood can be prevented, and therefore more investment in policies and programs that effectively improve child well-being should be a priority. Considering CRP levels in children’s clinical evaluation, and after excluding an acute infection, it can be useful to signal possible situations of threat for the child that may warrant further investigation. Further research is still needed to better understand the mechanisms explaining the emergence and persistence of health poorer outcomes later in life for victims of abuse. Thus, efforts focusing on preventing, identifying and stopping ACEs exposure and bullying victimization should be in place to better protect children. When prevention of victimization is no longer possible, efforts should be made to mitigate the health consequences.
